# Crim1 inhibits angiotensin II-induced hypertrophy and preserves Kv4.2 expression in cardiomyocytes

**DOI:** 10.22038/IJBMS.2022.61459.13602

**Published:** 2022-10

**Authors:** Jionghong He, Guiling Xia, Long Yang, Zhi Jiang, Ying Yang, Zhaomei Huo, Chuxian Guo

**Affiliations:** 1 Department of Cardiology, Guizhou Provincial People’s Hospital, Guiyang 550002 China; # These authors contributed eqully to this work

**Keywords:** Angiotensin II, Cysteine-rich transmembrane, bone morphogenetic protein - regulator 1, Ion channel remodeling, Transient outward - potassium current, Ventricular hypertrophy

## Abstract

**Objective(s):**

Angiotensin II (Ang II) plays a key role in the regulation of myocardial hypertrophy via downstream cysteine-rich transmembrane bone morphogenetic protein regulator 1 (Crim1). However, it is still unclear whether Crim1 is involved in ionic channel remodeling. The study aimed to explore the effects of Crim1 on transient outward potassium current (Ito) and Kv4.2 (the main subunit of I_to _channel) expression in hypertrophic ventricular cardiomyocytes.

**Materials and Methods:**

The ventricular cardiomyocytes were isolated from the neonatal rats. Hypertrophy was induced by Ang II. Crim1 expression was modulated by using adenovirus transfection. The expression of myosin heavy chain beta (β-MHC), Crim1, and Kv4.2 was determined by RT-qPCR and western blot. The cellular surface area was assessed using Image J software. I_to_ was recorded by the whole-cell patch clamp technique.

**Results:**

Ang II-induced hypertrophy in cardiomyocytes was identified by their larger cellular surface area and higher mRNA expression of β-MHC. Ang II significantly decreased the expression of Crim1 and Kv4.2 and reduced I_to_ current density. However, Crim1 overexpression abolished the Ang II-induced hypertrophy and preserved the expression of Kv4.2 and I_to_ current density.

**Conclusion:**

Crim1 overexpression inhibits Ang II-induced hypertrophy and preserves I_to_ current density via up-regulating Kv4.2 in ventricular cardiomyocytes from neonatal rats. Crim1 could have a role in the development of ventricular arrhythmia in hypertrophic hearts.

## Introduction

Ionic channel remodeling is an essential pathophysiological process in patients with cardiac hypertrophy (1, 2). Transient outward potassium current (I_to_) is involved in phase 1 of the action potential. It regulates the voltage-gated Ca^2+^ channel and balances the inward and outward currents following phase 2. In hypertrophic cardiomyocytes, ionic channel remodeling is remarkable in the dysregulation of Ito channels, leading to delayed repolarization and prolonged action potential duration (APD), which is considered the electrophysiological mechanism of malignant ventricular arrhythmia (3-5).

Ito channel consists of a pore-forming α subunit and an auxiliary β subunit. The α subunit has a fast and a slow component. The former is assembled by Kv4.2 and Kv4.3 subunits. Previous studies found that Kv4.2 is the major functional subunit in regulating the Ito current in rodents (6-9). 

Angiotensin II (AngII) through angiotensin receptor type 1 (AT1) is the main signaling pathway that leads to pathological cardiac hypertrophy (10, 11). In cultured atrial cardiomyocytes, AngII induced reduced I_to_ density, which was blocked by the AT1 antagonist, losartan (12).

Bone morphogenetic proteins (BMPs) are members of the transforming growth factors-β (TGF-β) superfamily. The expression of BMP4 is up-regulated in myocardial hypertrophy induced by pressure load and Ang II, the expression of BMP4 can induce cardiomyocyte hypertrophy, apoptosis, and myocardial fibrosis, and enhance the effect of myocardial hypertrophy induced by Ang II (13). Cysteine-rich transmembrane bone morphogenetic protein regulator 1 (Crim1) is a transmembrane protein and is widely expressed (14-17). Crim1 participates in vascular tube formation and heart development (14, 16, 18). Crim1 deficiency results in perinatal death with multiple organ defects (19, 20). The transmembrane structure of Crim1 is similar to the BMPs inhibitor chordin, which makes it become a regulatory molecule of the TGF- β subfamily (21, 22). Crim1 binds to BMP4 and BMP7 through CRR fragments and inhibits BMPs (23). 

Our previous study showed that Crim1, as a downstream signal of AT1, is involved in the negative regulation of ventricular cardiomyocyte hypertrophy in rats (24). However, it is unclear whether Crim1 is involved in I_to_ channel remodeling in hypertrophic ventricular myocytes. The study aimed to clarify the regulatory effect of Crim1 on Kv4.2 expression and I_to_ in hypertrophic ventricular cardiomyocytes from neonatal rats.

## Materials and Methods

***Isolation and culture of neonatal rat ventricular myocytes***

Neonatal Sprague-Dawley rats at 1-day old were purchased from the Animal Center of Nanjing Jiangning Qinglongshan [license number: SCXK (Su) 2017-0001]. Cardiomyocytes were isolated from ventricles as described (25). Briefly, the rats were sacrificed after anesthesia. Rat ventricles were digested with 0.01% trypsin (Sigma, USA) and 0.03% type II collagenase (Sigma, USA), followed by differential adhesion and 5-bromo-2-deoxyuridine (5-BrdU; Gibco-BRL, USA) affinity purification. The cells were cultured in high-glucose Dulbecco’s modified eagle medium (DMEM; Gibco-BRL, USA) containing 10% fetal bovine serum (FBS; Gibco-BRL, USA) at 37 °C in a 5% CO_2_ incubator. 48 hr later, the cells were cultured in serum-free DMEM with high glucose for the next experiment.

***Identification of cardiomyocytes***

Cells were cultured on fibrin (Sigma, USA) coated glass slides for 48 hr and α-striated muscle sarcomere actin (α-SCA) was detected by immunofluorescent staining. Neonatal rat cardiac fibroblasts were used as control. 

***Cell transfection***

Cell transfection followed the protocol in our previous study(24). Briefly, the primary ventricular myocytes were transfected using the recombinant adenovirus expressing Crim1 [Ad-Crim1; the Crim1 adenovirus expression vector was constructed by subcloning rat Crim1 (NM_001169103) coding sequence into adenovirus shutter vector, Thermo Fisher Scientific, Inc.]. The multiplicity of infection (MOI) of Ad-Crim1 was 100 (active viral particles per myocyte). Empty virus (Thermo Fisher Scientific, Inc.) at MOI=100 served as control. 

***Grouping and interventions***

Cardiomyocytes were divided into four groups: (1) In the control group, Ad-null was added. After 6 hr, the cells were incubated in two volumes (6 milliliters) of fresh serum-free DMEM for 48 hr. (2) In the Ang II group, Ad-null was added. After 6 hr, the cells were incubated in two volumes of fresh serum-free DMEM with a total of 0.1 μM of angiotensin II (Ang II; Gibco-BRL, USA) for 48 hr. (3) In the Crim1 group, Ad-Crim1 was added. After 6 hr, the cells were incubated in two volumes of fresh serum-free DMEM for 48 hr. (4) In the Crim1+Ang II group, Ad-Crim1 was added. After 6 hr, the cells were incubated in two volumes of fresh serum-free DMEM with a total of 0.1 μM of Ang II for 48 hr.

***Determining the effectiveness of the intervention on hypertrophy in cultured cells***

Hypertrophy of the cardiomyocytes was identified by the β-myosin heavy chain (β-MHC) mRNA expression and cell surface area (26). Cells were cultured on glass slides. Crystal violet staining assay was performed. Twenty fields of view were randomly selected. The cellular surface area was assessed using Image J software.

***RT-qPCR***

Total RNA was extracted using the RNeasy Mini Kit (Qiagen, China) according to the manufacturer’s instructions. A total of 1 µg of RNA was reverse transcribed using random hexamers from a first-strand cDNA synthesis kit (Qiagen, China). The mRNA expression was measured by the RT-qPCR method. The cycling conditions were as follows: 95 °C for 10 min, followed by 40 cycles of 15 sec at 95 °C and 30 sec at 60 °C. The mRNA levels were normalized to glyceraldehyde-3-phosphate dehydrogenase (GAPDH) and determined using the 2-ΔΔCq method. Primers were designed using Oligo 6.0 software. The sequence of the primers were shown as follows: β-MHC (sense primer: 5’- CAAGGAGCTCACCTACCAGA -3’; antisense primer: 5’- CAGCTTGTTGACCTGGGAC T-3’), Crim1 (sense primer: 5’- GTCTTTCCCCGGCGATCA -3’; antisense primer: 5’- TTGTTGCAGGTTCGGATGGT -3’) Kv4.2 (sense primer: 5’- GCTCTTCAGCAAGCAAGTTC -3’; antisense primer: 5’- TCCGACTGAAGTTAGACACG -3’) and GAPDH (sense primer: 5’- GTCAGTGGTGGACCTGGACCT -3’; antisense primer: 5’- AGGGGAGATTCAGTGTGGTG -3’). The results of each mRNA were obtained by detecting three groups of different samples, and each sample was repeated three times.

***Western blotting***

Total protein (40 μg) was loaded and then transferred to a nitrocellulose membrane after electrophoresis. The membrane was blocked with 5% skim milk for 1 hr. Rabbit anti-rat GAPDH antibody (1:1000; Shanghai Kangcheng Biotech, China), rabbit anti-rat ***α-SCA*** antibody (1:100; Sigma), rabbit anti-rat α striated muscle sarcomere actin (α-SCA) antibody (1:100; Sigma), rabbit anti-rat Crim1 antibody (1:100; Beijing Boao Sen Company, China), or rabbit anti-rat Kv4.2 antibody (1:1000; Abcam, USA) were added before overnight incubation at 4 °C. After thorough rinsing, horseradish peroxidase-labeled secondary antibody (1:3000; Santa Cruz, USA) was added for incubation at room temperature for 1 hr. Protein bands were detected with a Bio-Rad chemiluminescence detector and analyzed with Image J software. The results of each protein were obtained by detecting three groups of different samples.

***Whole-cell patch clamp detection***

A glass microelectrode (Beijing Zhengtianyi Electronics, China) formed a high resistance seal with the cells and ruptured the membrane. I_to_ was recorded under the voltage clamp mode. Current density analysis was used (current density [pA/pF]=current intensity/capacitance) to avoid errors caused by cell size. The action potential of the individual cells was recorded under the current clamp mode. The current signal was guided by an Ag/AgCl electrode and amplified by a patch clamp AXON 700B amplifier (Axon, USA)), through an AD/DA converter board, and stored in a computer hard disk. During the experimental procedure, stimulation discharge, and signal acquisition were controlled by pCLAMP 10.0 software. In the I_to_ depolarization step, the clamping voltage was set to −80 mV with an −40 mV to +70 mV pulse stimulation series, with a step voltage of 10 mV, wave width of 300 ms, and frequency of 0.2 Hz. 

***Statistical analysis***

Statistical analysis was performed using SPSS 19 software. All data are expressed as mean ± SD. Differences among groups were compared by one-way analysis of variance, and the q test was used for comparison between groups. A *P*-value of < 0.05 was considered statistically significant.

## Results

***Cardiomyocyte identification***

**Figure 1:**
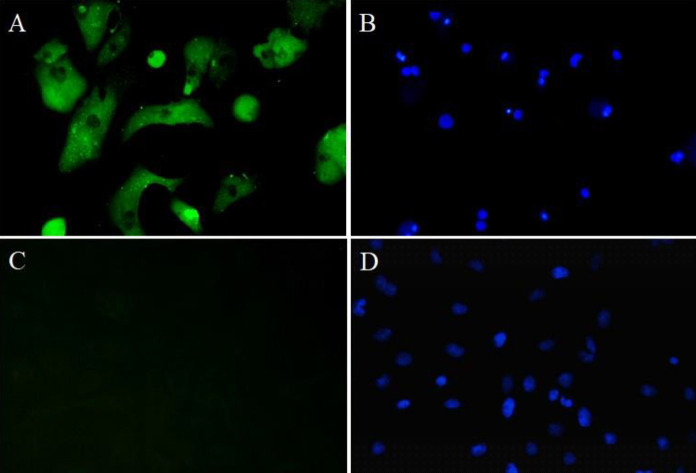
Identification of ventricular cardiomyocytes. Representative pictures of the immunofluorescent staining in the isolated cardiomyocytes. A: Cardiomyocytes showed green fluorescence targeting the α-striated muscle sarcomere actin (α-SCA)

Immunofluorescent staining of α-SCA was performed 48 hr after cell isolation to identify cardiomyocytes (Figure 1). The percentage of α-SCA-positive cells was 93.7%.

***Effects of Ad-Crim1 transfection***

**Figure 2.**
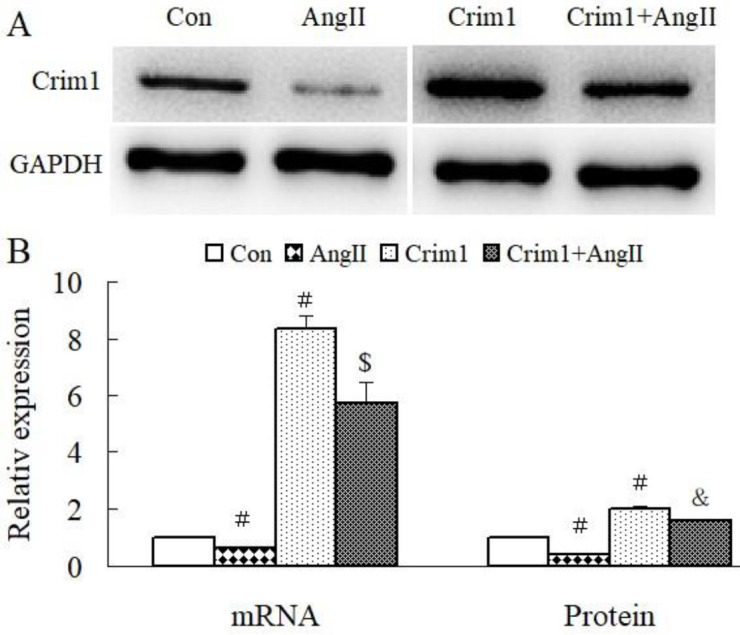
Effects of angiotensin II and Ad-Crim1 treatment on mRNA and protein expression of Crim1 in ventricular myocytes. A: Western blotting of Crim1. B: Semiquantitative analysis of Crim1 mRNA and protein expression among groups. # *P*<0.01 vs Con group; $*P*<0.05, & *P*<0.01 vs Ang II group

Compared with the control group, the Ang II group was lower in mRNA and protein expression of Crim1 (Figure 2). Ad-Crim1 transfection significantly increased the expression of Crim1 independent of the presence of Ang II (Figure 2).

***Effects of Crim1 overexpression on Ang II-induced cardiomyocytes hypertrophy***

**Figure 3:**
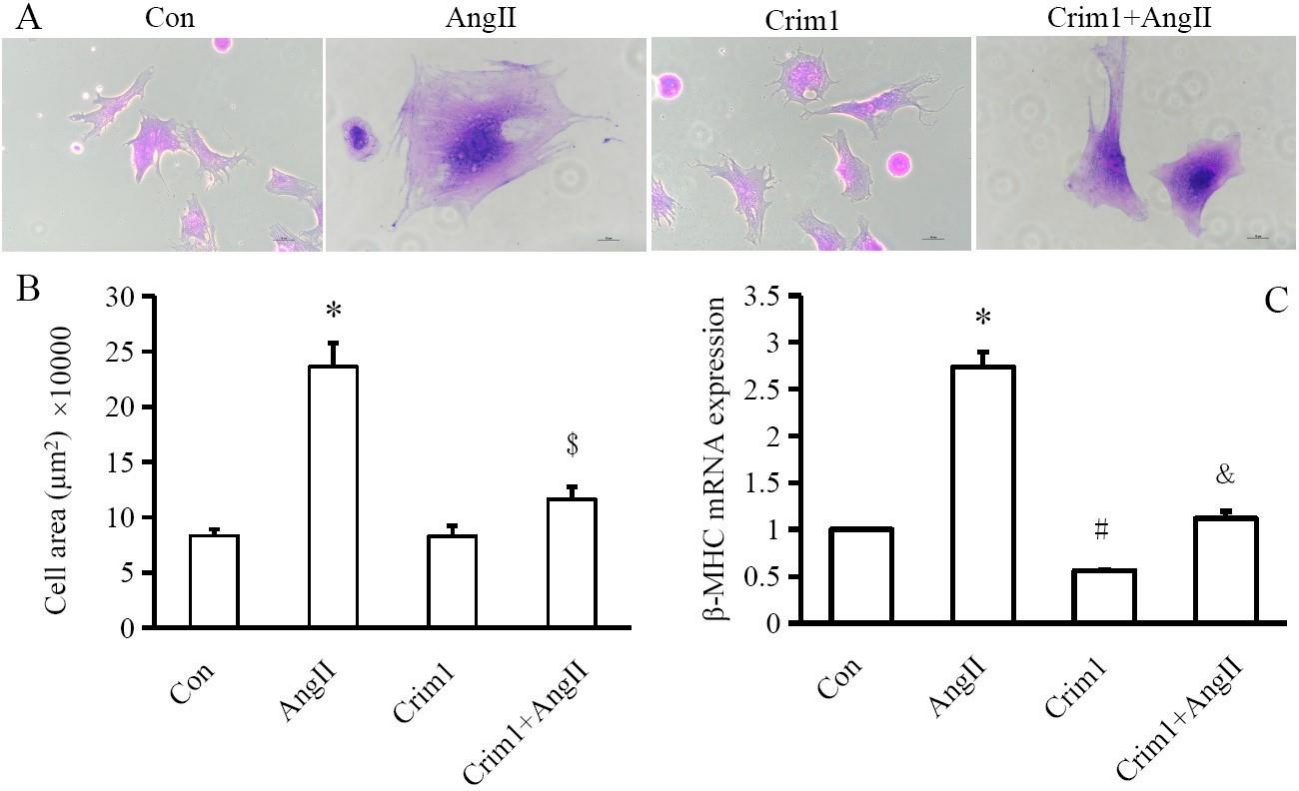
Angiotensin II stimulation promoted the β-MHC mRNA expression and increased cell surface area in ventricular myocytes which was attenuated by overexpression of Crim1. A: Representative pictures of crystal violet staining in the ventricular cells from the four groups. B: Quantitative analysis of cellular surface area among groups. C: Quantitative analysis results of β-MHC mRNA expression. *P<0.05, # *P*<0.01 vs Con group; $*P*<0.05, &* P*<0.01 vs Ang II group

Compared with the control group, the Ang II group had significantly larger surface areas of cardiomyocytes and higher mRNA expression of β-MHC, indicating the cardiomyocyte hypertrophy induced by Ang II. In the Crim1+Ang II group, the cardiomyocyte hypertrophy was not significantly different from the control group but was attenuated as compared with the Ang II group, indicating that overexpression of Crim1 inhibited the Ang II-AT1 signaling pathway in cardiomyocyte hypertrophy (Figure 3).

***Effects of Crim1 overexpression on Kv4.2 expression in cardiomyocytes***

**Figure 4:**
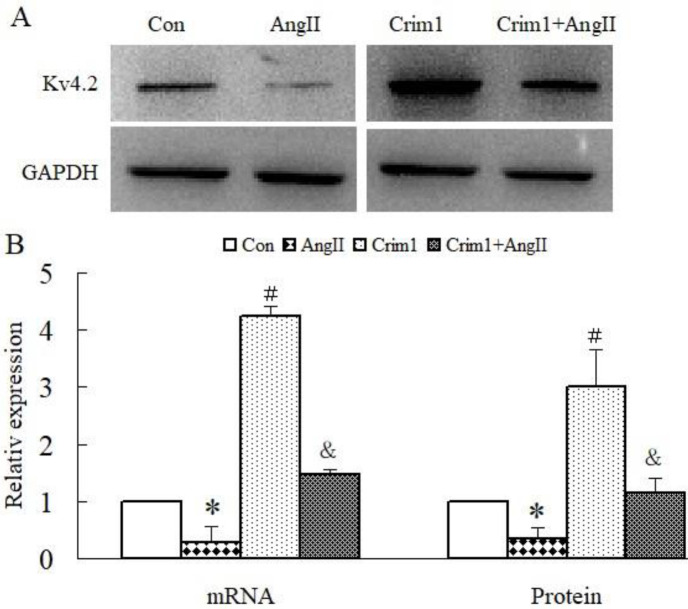
Angiotensin II inhibited the protein and mRNA expression of Kv4.2 which was attenuated by overexpression of Crim1. A: Western blotting of Kv4.2 protein. B: Semiquantitative analysis of Kv4.2 mRNA and protein expression among groups. *P<0.05, # *P*<0.01 vs Con group; $*P*<0.05, & *P*<0.01 vs Ang II group

In the Ang II group, the expression of Kv4.2 was significantly decreased in both mRNA and protein levels in comparison with the control group. In contrast, the expression of Kv4.2 in the Crim1+Ang II group was significantly higher than that in the Ang II group (Figure 4). 

***Effects of Crim1 overexpression on I***_to_ ***in cardiomyocytes***

**Figure 5:**
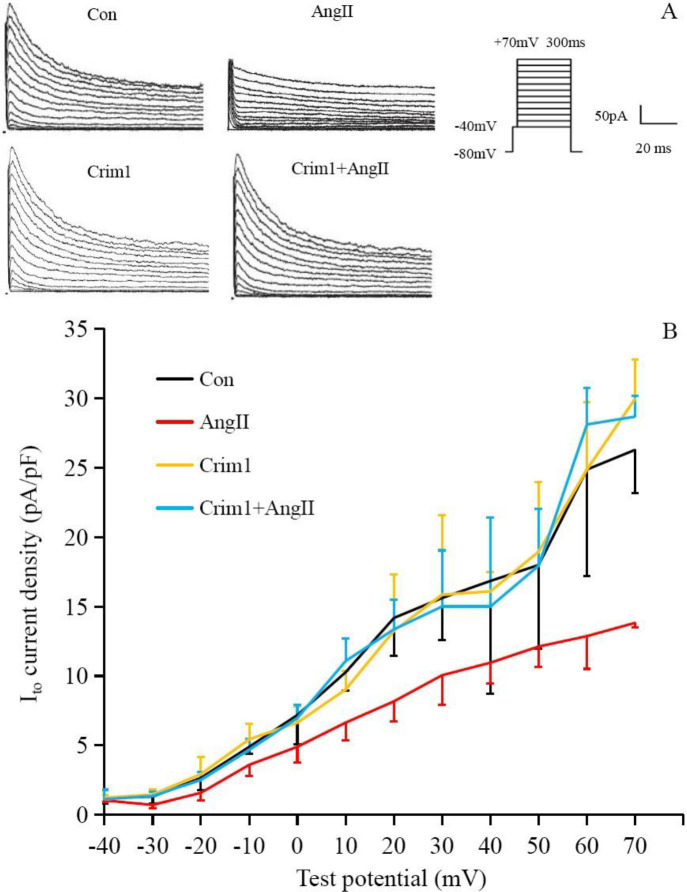
I_to_ densities in ventricular myocytes. A: Typical I_to_ currents were recorded in the cardiomyocytes from the four groups. B: Current-voltage (I-V) curve relations. At a stimulation voltage of -20–+70 mV, angiotensin II significantly decreased I_to_ density (*P*<0.05), which was markedly attenuated by overexpression of Crim1 (*P*<0.05; cell numbers are 6, 6, 3, and 3 in Con, Ang II, Crim1, and Crim1+AngII groups, respectively)

At a stimulation voltage of -20 - +70 mV, I_to_ current density in the Ang II group was significantly lower than that in the control group, and the peak current density was decreased by 47.5% (Ang II group vs. control group, *P*<0.01). The Crim1+Ang II group had significantly higher I_to_ current density than the Ang II group (*P*<0.05) (Figure 5). 

C: Cardiac fibroblasts were negative for fluorescence. B and D: Blue fluorescent revealed the nuclei. A and B, C and D are the same view, respectively. (×200)

β-MHC: β-myosin heavy chain; Ad-Crim1: Recombinant adenovirus expressing Crim1; Con: Control; Ang II: Angiotensin II

β-MHC: β-myosin heavy chain; Ad-Crim1: Recombinant adenovirus expressing Crim1; Con: Control; Ang II: Angiotensin II

β-MHC: β-myosin heavy chain; Ad-Crim1: Recombinant adenovirus expressing Crim1; Con: Control; Ang II: Angiotensin II

β-MHC: β-myosin heavy chain; Ad-Crim1: Recombinant adenovirus expressing Crim1; Con: Control; Ang II: Angiotensin II

## Discussion

In our study, Ang II stimulation resulted in significant down-regulation of Crim1 and Kv4.2, and a reduction in I_to_ current density in the ventricular cardiomyocytes *in vitro*. Crim1 overexpression abolished the Ang II-induced cardiomyocyte hypertrophy, the down-regulation of Kv4.2, and the reduction of I_to_ current density. The results showed that Crim1 plays a negative regulatory role on Kv4.2 expression and I_to_ current density in cardiomyocytes.

Ang II, the main active factor of the angiotensin system, is a pivotal endogenous cytokine leading to pathological myocardial hypertrophy through AT1 (10, 11). A lot of signals regulated the myocardial hypertrophy as the down-stream of AT1, such as tumor necrosis factor (TNF)-á (27), secretory-leukocyte-protease-inhibitor (SLPI) (28), Monocyte chemoattractant protein-1 (MCP-1) (29), nuclear factor-kappaB (NF-kappaB) (30), TGF-â (31), and calcineurin (32). Our previous study found that Crim1, as a downstream signal of AT1, suppresses ventricular myocardial hypertrophy both *in vitro* and *in vivo* (24). In the current study, Ang II stimulation resulted in significant ventricular cell hypertrophy and decreased the mRNA and protein expression of Crim1. Overexpression of Crim1 by Ad-Crim1 transfection significantly attenuated the effects of Ang II on ventricular cell hypertrophy. The result indicated the negative regulatory role of Crim1 on Ang II-induced cardiomyocyte hypertrophy.

The Angiotensin system is involved in the regulation of the structure and function of ion channels on cardiomyocytes. Iravanian *et al*. (33) reported that connexin43 (Cx43) was down-regulated in angiotensin-converting enzyme (ACE) gene knock-in mice. Captopril or losartan up-regulated Cx43 protein expression and phosphorylation ratio, resulting in the reduction of ventricular tachycardia incidence. Moreover, a study (34) indicated that cardiac-specific ACE overexpression in mice resulted in changes in connexins consistent with the phenotype of low-voltage electrical activity, conduction defects, induced ventricular arrhythmia, and higher cardiogenic mortality. Tyan *et al*. (12) showed that in atrial myocytes, a short-term (2 hr) treatment with Ang II significantly reduced I_to_ density. This effect was prevented by a 30-min pretreatment with losartan, a selective antagonist of AT1.

However, only a few reports on the specific molecular mechanisms of Ang II and AT1 are involved in ion channel remodeling in cardiomyocytes. He *et al*. (32) reported that the activation of AT1 by mechanical stretch in neonatal myocytes resulted in the remodeling of the inward rectifier potassium (I_k1_) channel and the change in APD by activating calcineurin-nuclear factor of activated T-cells (NFAT) signaling pathway. Gou *et al*. (35) demonstrated that protein kinase Cε (PKCε) isoenzyme mediates the inhibitory action of Ang II on delayed rectifier K+ current (I_ks_) and by phosphorylating distinct sites in KCNQ1/KCNE1 (two encoding genes for I_ks_ ion channel), conventional PKC and PKCε isoenzymes produce the contrary regulatory effects on the channel. Binas *et al*. (36) confirmed that, in ventricular muscle, a stimulus induced increase in MicroRNA-221/222 *in vivo* by Ang II, leading to down-expression in proteins of CACNA1c and KCNJ5, the encoding genes of L-type calcium channel and inwardly rectifying potassium channel, respectively, and attenuation of ion currents of the two channels.

I_to_ is an outward potassium current that slows the repolarization of the action potential in phase 1 of the action potential. In hypertrophic cardiomyocytes, the expression of Kv4.2 and Kv4.3 is down-regulated and the density of I_to_ is reduced. It leads to abnormal repolarization and prolongation of APD, which causes fatal ventricular arrhythmias (37-40). We found that Crim1 inhibits the reduction of Kv4.2 expression and I_to _current density induced by Ang II, indicating that Crim1 may be a regulator of ventricular arrhythmia in pathological hypertrophic hearts.

***Limitations***

The substance of the quantities of ion channels participating in ventricular electrical remodeling that affected the action potential of ventricular myocytes. This study only examined I_to_ and the mRNA and protein expression of kv4.2, and the action potential was not determined.

## Conclusion

Crim1 overexpression inhibits Ang II-induced hypertrophy and preserves I_to_ current density via up-regulating Kv4.2 in ventricular cardiomyocytes from neonatal rats. Crim1 could have a role in the development of ventricular arrhythmia in hypertrophic hearts.

## Authors’ Contributions

LY and JH Conceived the study or design. JH, YY, ZH, and CG Performed the experiments. JH and GX Analyzed data and prepared the draft manuscript. Z J and LY Modified the paper. LY critically revised the paper.

## Statement of Ethics

The experiments were approved by the Ethics Committee of Guizhou Provincial People’s Hospital (Hospital Ethics Review [2020] No. 076). 

## Conflicts of Interest

None.
